# Mini-review of kidney disease following hematopoietic stem cell transplant 

**DOI:** 10.5414/CN109276 

**Published:** 2018-03-26

**Authors:** Ramy Sedhom, Daniel Sedhom, Edgar Jaimes

**Affiliations:** 1 Rutgers Robert Wood Johnson Medical School, Department of Internal Medicine, New Brunswick, NJ, and; 2 Memorial Sloan Kettering Cancer Center, Department of Medicine Renal Service, New York, NY; Renal Division, Weill-Cornell Medical College, New York, NY, USA

**Keywords:** hematopoietic stem cell transplantation, chronic kidney disease, acute kidney disease, renal complications

## Abstract

Advancements in hematopoietic cell transplantation (HCT) have broadened indications for its use and resulted in more long-term survivors. Stem cell transplantation is associated with several well-known toxicities, although renal complications are not well defined. Acute and chronic kidney disease remains a common complication following transplantation itself. Incidence and risk factors for the development of chronic kidney disease (CKD) is less well understood. Recent estimates suggest that nearly 15% of subjects undergoing HCT will develop CKD, a complication that can progress to end-stage renal disease (ESRD), disrupts overall quality of life, and reduces overall survival. Several commonly-reported risk factors include acute kidney injury, graft-versus-host disease, and long-term calcineurin inhibitor use. This review highlights the incidence, timeline, etiology, risk factors, and prognosis of kidney disease in the setting of hematopoietic stem cell transplantation. Investigation of the causes of CKD is needed, as are ways to prevent, mitigate, and treat kidney injury. Renal disease importantly reflects prognosis, with dialysis-requiring patients carrying greater than 80% mortality after 3 years. Although CKD following HCT is common, prospective studies are needed to confirm risk factors and better define the underlying mechanisms in order to promote therapies that prevent this complication.

## Introduction 

Chronic kidney disease (CKD) is being increasingly recognized as a global health concern. Related outcomes include progression to end-stage renal disease (ESRD), complications of reduced kidney function, and increased risk of other chronic disease states such as cancer, infection, and cardiovascular disease [1]. Patients with CKD are more likely to die than to develop kidney failure. Hematopoietic cell transplantation (HCT) patients who develop CKD thus carry a high risk for death, cardiovascular events, and repeat hospitalizations [1, 2]. This review will describe some of the unique presentations and proposed etiologies of kidney injury, the clinical outcomes, and suggested management of the kidney disorders following hematopoietic cell transplantation. 

HCT provides radical treatment opportunities for several malignant and nonmalignant disorders otherwise incurable. HCT has contributed greatly to improving quality of life and survival of patients with previously terminal diseases [3]. Despite advances in supportive care, transplant-related organ dysfunction remains a major obstacle. Major complications following transplant include immunologic toxicity, opportunistic infections, and organ complications including hepatic, cardiovascular, and pulmonary toxicities. Acute kidney injury (AKI) is common in transplanted patients, is not well understood, and has significant associated morbidity and mortality [4, 5, 6, 7, 8, 9, 10, 11, 12, 13, 14, 15]. Kidney injury may be caused by the conditioning chemotherapy regimen, total-body irradiation, use of nephrotoxic agents, infection, hepatic sinusoidal obstruction syndrome (veno-occlusive disease of the liver), transplantation-associated microthrombotic angiopathy, and graft-versus-host disease (GVHD) [16, 17, 18, 19]. Those who overcome AKI and survive develop long-standing renal complications within several years [20, 21, 22, 23, 24, 25]. CKD ultimately develops in most patients. 

CKD has also been described following liver, cardiac, and heart-lung transplants. The percentage of patients with chronic kidney disease and ESRD are similar to patients with HCT [26]. However, the risk factors for development of CKD vary by the type of transplant. In liver transplant patients, pre-existing diabetes, female gender, and hepatitis are unique risk factors. In cardiac patients, hypertrophic cardiomyopathy, African American race, and history of a previous transplant are unique risk factors. In heart-lung transplant, hypertension and serum creatinine appear to be the most important risk factors. Risk factors in the hematopoietic stem cell transplant will be discussed in detail and include GVHD, AKI, and long term calcineurin inhibitor use [26]. 

## Hematopoietic stem cell transplantation – background 

Hematopoietic stem cell transplantation has become an effective and increasingly used treatment since the 1980s. Every year, tens of thousands of patients worldwide undergo stem cell transplant to treat malignant and nonmalignant diseases. Transplant regimens involve either myeloablative or reduced-intensity conditioning; haploidentical or mismatched donors; cells from bone marrow, cord blood, or stem cells from peripheral blood; and immunotherapy to prevent graft vs. host disease (Table 1). Although long-term survivor rates have improved, advances in clinical care have led to a greater number of HCT recipients facing long-term complications. The complications are associated with their underlying disease, HCT preparation regimens, the transplant itself, or post-HCT diseases, infections or medication related toxicities. Included in this list and described in this review is the development of CKD. Although changes in kidney function are expected in both allogeneic and autologous transplants, the risk of both acute and chronic kidney injury are greater following allogeneic transplantation [27]. 

## Definition and epidemiology of kidney injury post-transplantation 

Renal failure was initially defined as a doubling of the serum creatinine within the first 100 days after transplant. It was first recognized as a major complication in transplant by Zager et al. [28, 29]. More recently, other criteria have been proposed that better characterize risk, injury, failure, and loss of kidney function. These include the RIFLE (risk, injury, failure, loss of kidney function, and end-stage kidney disease) system and the AKIN (acute kidney injury network) criteria [30]. 

Renal disease after HCT encompasses a wide spectrum of structural and functional abnormalities, including vascular (hypertension, thrombotic microangiopathy), glomerular (albuminuria, nephrotic glomerulopathies), and tubulointerstitial disease. All of these abnormalities may lead to a decreased glomerular filtration rate (GFR) and ultimately CKD. In the early post-transplant period, complications most commonly include renal insufficiency secondary to sepsis, hepatic sinusoidal obstruction syndrome, or medication-induced nephrotoxicity. In the intermediate period, AKI can be secondary to thrombotic microangiopathy and is accompanied by high mortality rates [5, 11]. By 3 – 6 months, most patients have developed kidney injury with reductions in GFR [31]. Six months after HCT, kidney injury is related to the nephrotic syndrome secondary to chronic GVHD, although the development of CKD at this time can be multifactorial in origin [32, 33, 34]. The development of kidney injury interferes with adequate dosing of important immunosuppression, and this may then predispose to other major organ dysfunctions, such as lung and liver failure, and ultimately transplant rejection. Thus, unsurprisingly, the appearance of AKI is associated with decreased overall survival. In patients requiring dialysis, mortality ranges from 55 to 100% [35, 36, 37]. Overall, patients who undergo HCT experience a greater fall in their glomerular filtration rate than age-related cohorts [38]. 

## Pathogenesis of kidney injury in transplantation 

Causes of kidney injury in transplant patients are often multifactorial and may not lead to organ biopsy if acute, self-limited, or slowly developing. Although case reports, small single-center case series, and autopsy studies are available for review, most focus on a single pathologic cohort and under­appreciate the potential for overlap of several disease states. Nonetheless, several major risk factors have been identified. They will be discussed and separated into acute and chronic kidney disease. 

### Acute kidney injury 

Acute kidney injury in the setting of HCT is precipitated by common causes of injury, similar to those seen in the intensive care unit. Acute GVHD, hepatic sinusoidal obstruction syndrome, calcineurin inhibitors, and viral infections are unique to this population and often unique in their presentation (Table 2). Acute GVHD is mediated by cytokine release and driven by cytotoxic T lymphocytes. Clinical presentation includes skin changes, gastrointestinal manifestations, and symptoms related to liver injury [39]. The effects of GVHD on the kidney have only recently been appreciated as a cause of kidney injury [40]. Because the effects are thought to be more chronic over time, it will be further discussed under causes of CKD. 

Hepatic sinusoidal obstruction syndrome is a unique cause of AKI, usually encountered in the first 30 days after transplant. It is mediated by endothelial cell injury of hepatic venules secondary to high-intensity conditioning regimens. Symptoms are often a manifestation of underlying hepatic sinusoidal thrombosis and portal hypertension [41]. Clinical presentation includes fluid retention, hepatomegaly, right upper quadrant pain, and ascites. Transaminases, bilirubinemia, and low urine sodium can be seen in laboratory studies [41, 42, 43]. These signs and symptoms precede the development of renal insufficiency. Up to 80% of patients with veno-occlusive disease (VOD) will develop AKI [41, 42, 43]. Risk factors include advanced age, pre-existing hepatic disease, CMV seropositivity, and medications with liver toxicity [44]. Treatment is largely supportive with diuretics, analgesics, paracentesis, and dialysis if needed. 

Calcineurin inhibitors (cyclosporine, tacrolimus) are used for prophylaxis against GVHD and were initially thought to be the major cause of acute injury after hematopoietic cell transplantation. They are combined with methotrexate in myeloablative allogeneic transplant patients and with steroids in nonmyeloablative transplantation. Several studies have brought to question the significance of cyclosporine on the development of acute kidney injury [10, 45, 46]. Cyclosporine has a narrow therapeutic index and requires adjustment based on elevations in serum creatinine. It is statistically difficult to determine the contribution of cyclosporine to renal dysfunction as several toxic insults usually occur concurrently (sepsis, GVHD, medication, other causes of nephrotoxicity). Reductions in renal blood flow may impair endothelial cell function leading to decreased production of vasodilators and enhanced release of vasoconstrictors. This may explain clinically the development of AKI [46]. 

Chronic cyclosporine therapy in solid-organ transplantation has been associated with the development of nephrotoxicity. Long-term complications are unusual following HCT because it is given in full doses for only several months. The factors responsible for chronic cyclosporine nephrotoxicity are not well understood, and several authors implicate genetic predispositions [47]. Characteristic vascular and interstitial changes include obliterative ateriolopathy, glomerular scarring, and focal segmental glomerulosclerosis [47]. Considering the importance of cyclosporine for the success of HCT, its nephrotoxic potential should be recognized. Further studies are needed to evaluate for drug synergism, especially when considering other nephrotoxic medications frequently administered such as vancomycin, aminoglycosides, and amphotericin therapy. 

In appropriately-selected patients, T-cell depleted (TCD) HCT has shown similar survival and disease-free survival as in those patients with conventional HCTs [48]. TCDs have the potential to decrease the risk of renal impairment, as they obviate the need for calcineurin inhibitors (CNIs). In multivariate analysis, TCDs offered better renal outcomes, particularly important considering the increased risk of all-cause mortality. 

## Chronic kidney disease 

### Graft-versus-host disease 

Although traditionally not believed to involve the kidney, recognition for GVHD as a potential cause of renal dysfunction post-transplant is growing [49, 50, 51]. The relationship at this time is extremely difficult to ascertain as histological diagnosis of renal GVHD is difficult to make, and no formal pathologic criteria exist. The mechanism proposed by the National Cancer Institute consortium involving CKD is endothelial injury, leading to inflammation, cytokine cascades by tumor necrosis factor, interleukin 6, and transforming growth factor-β (TGF-β). Fibrosis and organ failure later result following immune intolerance of chronic GVHD [52]. Infiltration of CD3+, CD4+, and CD8+ cells and CD68+ macrophages into the renal interstitium have also been described in rat models. Peritubular capillaritis, tubulitis, glomerulitis, and endarteritis were observed with worsening acute GVHD suggestive of an inflammatory process. These findings were thought to be driven by cytokine release or direct targeting of a T-cell-mediated cascade [53, 54, 55][Table Table3]. 

### Hematopoietic cell transplantation-associated thrombotic microangiopathy 

Thrombotic microangiopathy (TMA) includes a spectrum of clinical diseases characterized pathologically by severe endothelial damage and platelet aggregation. It is a major compilation of HCT, usually presenting 6 – 12 months after HCT [9, 11]. The condition, formerly known as bone marrow transplant nephropathy, was renamed transplant-associated thrombotic microangiopathy (TA-TMA) per consensus criteria [56]. Risk factors include treatment with total-body irradiation, the use of CNIs, the combined use of sirolimus and tacrolimus, acute GVHD, infection, and transplant of peripheral blood stem cells [9, 57]. Clinically, it is characterized by thrombocytopenia, hemolytic anemia, neurologic disturbances, and renal impairment. The end result is endothelial dysfunction and ischemic organ injury. 

Disease is typically apparent in the first 20 days post-transplant [58]. Diagnosis is increasingly difficult as patients have several reasons for anemia, thrombocytopenia, renal dysfunction, fever, and neurologic abnormalities, including delayed engraftment, infection, medication effect, or GVHD. In addition, recent studies have shown that many patients may not fulfill clinical criteria of TMA based on laboratory findings, despite confirmation of pathologic changes [59, 60, 61]. 

The incidence varies greatly and is limited by diagnostic criteria and the difficulty in obtaining renal biopsies immediately following HCT. Histologic analysis of the kidney reveals necrotizing arterial and glomerular lesions, and thrombi within the glomerulus and renal arterioles [61]. Endothelial damage is thought to occur by activation of the coagulation system, leading to formation of thrombin and the deposition of fibrin [62]. Investigation into the coagulation cascade and complement system found deletions in genes encoding complement factor H and autoantibodies that likely contribute to the microangiopathy [60, 63, 64]. Diagnosing TMA remains difficult. In recent years, two consensus statements have been published in an effort to define clinical features and diagnostic criteria for post-transplantation TMA; even these two expert panels did not agree on all of the criteria [56, 65]. 

### Glomerular disease 

Membranous nephropathy (MN) is the most common glomerular disease in kidney biopsies in HCT recipients [66, 67]. Typical presentations include nephrotic syndrome or nephrotic range proteinuria, and near normal creatinine. Most patients are treated with immunosuppressive regimens, leading to complete resolution in 60 – 70% of cases [66, 67]. MN has been seen in biopsies together with focal segmental glomerulosclerosis, microaneurysms, global glomerulosclerosis, interstitial inflammation, interstitial fibrosis, acute tubular injury, tubular atrophy, and arteriosclerosis in HCT recipients [68]. This reflects the multiple injuries the kidney is subjected to and, in some instances, pre-existing disease in HCT patients. There has also been a frequent association of post-HCT MN with chronic GVHD, presenting as early as 2 months after transplantation, but more typically appearing 6 – 12 months later [69]. Very few cases of nephrotic syndrome after HCT have been reported in the absence of GVHD [50, 70, 71, 72]. 

Minimal change disease (MCD) is the second most common glomerular disease reported in post-HCT kidney biopsies. Similar to membranous nephropathy, patients present with nephrotic syndrome or nephrotic range proteinuria and near-normal creatinine. MCD in nontransplant settings has been associated with T-cell dysregulation or hypersensitivity reactions. In the setting of transplant, disease has been described in the absence of GVHD, distinguishing it from MN [71, 72]. Elevated levels of interferon-γ and tumor necrosis factor-α have been reported, suggesting a role for cytokine and antibody-mediated pathology [73, 74]. Case reports have also described class III lupus nephritis, focal segmental glomerulosclerosis, and IgA nephropathy [74, 75, 76]. 

### Infection 

HCT recipients are immunosuppressed hosts, even after marrow engraftment and tapering of immunosuppressive GVHD prophylaxis. Neutropenia secondary to chemotherapy and radiation increase the risk of bacterial, viral, and fungal infections. Compensatory mechanisms, such as cytokine-induced renal vasoconstriction and capillary leak during sepsis, are known to contribute to renal hypoperfusion. Complement-mediated renal injury has also been identified as a contributing source of AKI in the setting of sepsis. In addition, medications used to treat sepsis are nephrotoxic. Antifungal and aminoglycoside antibiotics are known nephrotoxins, but they are tubular rather than glomerular toxins [16, 77]. Amphotericin B is directly toxic to the distal tubular epithelium; it induces renal vasoconstriction, reducing renal blood flow and GFR. Several newer antifungal agents with less nephrotoxicity are available, and, therefore, use of amphotericin B should be limited whenever possible [78]. Aminoglycosides are also used both empirically and therapeutically. The nephrotoxicity of aminoglycosides is related to the intracellular accumulation of the drug resulting in disruption of membrane permeability and inhibition of intracellular phospholipases in proximal tubular cells [79]. In addition to bacterial, mycobacterial, and fungal organisms, transplant recipients are also susceptible to viral infections involving the kidney, which will be detailed here. 

### Viral nephropathy 

BK virus, a polyoma virus, occurs in normal hosts of all ages. The virus remains latent in the kidney and urinary tract and is activated following immunosuppression. Several groups have advocated for BK surveillance in HCT populations [80, 81]. Polyomavirus and adenovirus are also important causes of hemorrhagic cystitis. Clinical presentations vary and include hematuria, dysuria, and flank pain, with or without kidney injury. Histologic features include tubular epithelial intranuclear inclusions with granular texture, basophilic staining, sometimes surrounded by a clear halo, without cytoplasmic inclusions [80]. Risk factors for hemorrhagic cystitis include high levels of BK virus, receipt of cordblood or peripheral blood stem cell transplant, acute GVHD, and coinfection with other viruses [81, 82]. Early detection of BK nephropathy prior to renal damage reduces the rate of progression. Plasma levels of BK virus are more indicative of renal involvement than urinary levels [83]. In patients with BK viremia or evidence of nephropathy, first-line therapy is reduction of immunosuppression. Initiation of cidofovir or leflunomide should follow shortly thereafter [84, 85]. The definitive diagnosis of BK nephropathy requires a biopsy given the nephrotoxic effects of treatment. 

Adenovirus infection has long been recognized as a complication of HCT. Only recently has it been described as a cause of nephritis. Bruno et al. [86], reported an autopsy series of 21 cases of adenovirus nephritis in which most patients presented with AKI, often incorrectly attributed to other causes, such as drug toxicity, with delayed diagnosis and treatment. Adenoviral inclusions may be seen in renal tubular epithelial nuclei. The distinctive features of adenovirus, compared with CMV and BK virus, include necrosis of tubules and interstitium, often with surrounding granulomatous and neutrophilic inflammation [87, 88, 89]. Immunohistochemical studies are important to confirm adenovirus in biopsy samples. Adenovirus has been reported to be sensitive to both cidofovir and brincidofovir [87, 88, 89]. 

CMV end-organ disease in HCT recipients has been reduced by prophylactic antiviral therapy. Whereas CMV pneumonitis ranges from 1 to 30%, incidence of renal CMV infections is much lower. However, drugs used for CMV prophylaxis or therapy, such as foscarnet or gangcyclovir, have renal toxicity [90]. Histopathologically, CMV can be seen as “owl-eye” nuclear inclusions, accompanied by cytoplasmic inclusions, focally in tubular epithelial cells or podocytes. Immunohistochemical studies on biopsy tissue are again useful for specific confirmation [90, 91]. 

### Autologous vs. allogeneic hematopoietic cell transplantation 

Smaller decrements in kidney dysfunction have been described among autologous HCT patients when compared to allogeneic HCT patients [27]. This is likely due to several factors. Autologous transplant patients typically receive milder conditioning regimens and are therefore less likely to develop some of the complications related to these exposures. The incidence of AKI is lower (29% vs. 40%) [12, 31, 92]. Myeloablative autologous transplant has the lowest incidence of AKI (22%) [92, 93]. This difference can be attributed to the fact that autologous transplant recipients do not suffer from acute or chronic GVHD, which, as described above, exposes the kidney to both direct and indirect damage. They also do not require CNIs to prevent or treat chronic GVHD and are not at risk for CNI toxicity. Most cases of AKI occur within the first 100 days after transplant, with an earlier onset in myeloablative (7 – 40 days) compared with nonmyeloablative regimens (22 – 60 days) [94]. AKI in both cohorts predict subsequent development of CKD [94]. Autologous patients, for reasons unknown, rarely develop complications like sinusoidal obstruction syndrome (7% in autologous transplant vs. 54% in allogeneic transplant) [41, 42, 43].  

### Clinical presentation 

CKD after HCT may not be recognized early given competing clinical priorities. In addition, as a consequence of chronic illness, bodily creatinine production may decline, and serum creatinine may be interpreted as deceptively “normal”. Proteinuria may not be identified early as urinalysis is not a routine diagnostic test. Patients may have a long list of past and present medications. The time of presentation can often be a clue. Physical exam may show edema or hypertension. Absence of elevations in blood pressure suggest interstitial rather than glomerular disease. Proteinuria in the nephrotic range indicates nephrotic syndromes rather than thrombotic microangiopathy. Nephrotic range proteinuria associated with chronic GVHD may indicate membranous nephropathy. Although azotemia is non-specific, the rate of decline in GFR, if rapid, points to thrombotic microangiopathy, which is 10 times as fast as that of common causes of progressive kidney injury [79].  

### Management 

Cure of CKD following HCT is unlikely. If specific causes are identified, they should be addressed (Table 4). This includes use of nonessential medications. Discontinuing CNIs, although logical in some patients, is difficult as alternatives are limited in subjects with GVHD. Prednisone can and should be used to reverse MCD. Rituximab has been shown to suppress dysregulated B-cells in chronic GVHD in associated membranous nephropathy [45]. 

Recent consensus guidelines recommended screening urinalysis as part of the 80-day post-transplant evaluation. After this, screening should be performed yearly. If macroalbuminuria is present, patients should be monitored every 3 – 6 months [95]. Control of blood pressure is also an important aspect of post-transplant care. Consensus recommendations are to measure blood pressure at each visit, with a maximum interval of yearly [96]. Thresholds for treatment of elevated blood pressure follow those of the general population [97]. 

Antagonism of the renin-angiotensin system is indicated in hypertensive patients. They are effective in prevention, mitigation, and treatment of radiation nephropathy, and should be first-choice therapy for hypertension [98, 99, 100, 101]. Angiotensin-converting enzyme inhibitors have been used in rodent models and have resulted in less azotemia, lower blood pressures, decreased proteinuria, and long-term preservation of renal function [102]. The difficult question and an area of future research is when to intervene. Should patients start medications as a preventative measure, at the first sign of injury, or at some point following transplant to protect their kidneys? Anemia may occur and is disproportionately severe in relation to their renal function, and is thought to be linked to erythropoietin (EPO) deficiency [46]. Supplemental EPO is effective in its treatment. Unfortunately, progressive renal failure may occur despite best efforts, and survival on dialysis is very poor, likely due to comorbidities. 

## Prognosis and outcomes 

### Mortality 

Zager et al. [28, 29, 103] noted the significant differences in mortality in transplant-related kidney disease. Transplant-related mortality was 17% without renal insufficiency, 37% in nondialysis-requiring renal failure, and 84% in patients with a dialysis requirement. Subsequent studies have confirmed the association of kidney injury with increased mortality. The adjusted odds ratio of 6-month mortality with dialysis requiring renal failure was 6.8× higher following adjustment for demographic, clinical, and transplantation-related variables [31, 104, 105]. Although future studies are needed to better understand the underlying disease processes, poor outcomes are perhaps due to nonrenal toxicities secondary to systemic cytokine release syndromes and immune-mediated damage initiated by renal injury. Of particular interest is the observation that hepatic, pulmonary, and gastrointestinal toxicities increase as the severity of kidney injury increases [106]. Multiple studies have demonstrated that mortality rates increase as the severity of kidney injury increases. 

### Chronic kidney disease 

CKD can be defined by objective measures including an elevation of creatinine, a decreased GFR (< 60 mL/min/1.73m^2^), or by structural or functional abnormalities other than a decreased GFR such as microalbuminuria, albuminuria, or proteinuria. Prevention and appropriate treatment of CKD in long-term survivors of HCT is an important issue that requires appropriate management by internists, nephrologists, cardiologists, and transplant oncologists. One year after transplant, the GFR is decreased in many patients. The incidence of CKD in studies is varied, ranging from 7 to 48%. Kidney injury can occur acutely within the first 6 months after transplant or delayed as far as 10 years following transplantation [27]. Risk factors for kidney injury include, but are not limited to, AKI, GVHD, age greater than 45 years, baseline kidney disease, hypertension, and exposure to total-body irradiation [26]. Despite early recognition of patients at high risk for kidney injury, prevalence of CKD has not changed. Patients who develop CKD after HCT have a worsened mortality, even when controlled for other comorbidities. 

A remarkably high percentage of post-HCT patients have evidence of proteinuria and all stages of CKD. By 3 – 6 months after transplantation, the GFR decreases in most patients [16, 20, 21, 27, 105]. The etiology is multifactorial. AKI certainly contributes to long-term impairment secondary to failure in resolving renal structure and function adequately (OR 32.8, 95% CI 4.3 – 250, p = 0.005) [27, 45, 104, 107, 108, 109, 110]. In addition, AKI is associated with an increased risk for hypertension, which contributes to CKD development [27, 45, 104, 107, 108, 109, 110]. Other risk factors include acute and chronic GVHD, age greater than 45 years old, hypertension, survival more than 1-year post transplantation, and exposure to high-dose total body irradiation [20]. 

Long-term survivors of HCT develop ESRD as a higher rate than the general population and have worsened mortality [95, 96, 97, 98]. CKD is known to be a risk factor for chronic diseases such as cardiovascular disease, infection, cancer, and ESRD. The overall burden of CKD in HCT patients has troublesome medical and economic implications, especially as recipients of HCT have increased longevity with advancements in chronic disease management. Thus, prevention of kidney injury has the potential to prevent advanced CKD and its associated morbidity and mortality. 

## Future approaches: The role of elafin and areas of future studies 

There clearly are distinct entities that occur after HCT, although the potentially independent role of GVHD, chronic inflammation, and exposure to CNI warrant further investigation to their role in the development and progression of CKD. Although most patients following HCT develop microalbuminuria by day 100 after transplant, it is currently unclear if this is a marker of nephropathy or simply a product of endothelial dysfunction. Given the risks and difficulty in obtaining a renal biopsy in this population, novel serum and urinary markers are needed to diagnose, risk stratify, and guide management for acute and chronic kidney injury. 

Numerous approaches have been made to identify methods to accurately diagnose GVHD in a noninvasive fashion. Elafin was first identified as an accurate plasmatic biomarker of cutaneous GVHD, correlating with disease severity and reflective of prognosis [111]. Elafin, a serine protease inhibitor, is mostly produced by epithelial cells, and plays a role in both immunomodulation and regulation of cell proliferation [96]. More recently, it was found that urinary elafin may potentially serve as a marker of renal inflammation and injury, as higher levels are associated with an increased risk of micro and macroalbuminuria, AKI, CKD, and death after HCT [54, 55, 112]. Randomized trials are currently underway in patients undergoing renal transplant assessing the potential to mitigate ischemia-reperfusion injury [54]. Future prospective studies looking more closely at urinary elafin are certainly needed in HCT recipients. 

## Conclusion 

Assessment of the etiology, characteristics, incidence, and severity of renal failure following transplantation are important to consider in light of its poor prognosis. Mortality is clearly associated with the severity of renal injury, approaching greater than 80% when dialysis is required. While risk factors are numerous, prevention is key. Early goal-directed therapy to successfully treat sepsis can prevent severe renal injury. Further studies are needed to improve success against hepatic veno-occlusive disease and transplantation-associated microangiopathy. 

Strategies to reduce AKI following transplant are needed, and there is a noticeable absence of randomized trials. Patients undergoing HCT, who survive and develop a drop in their eGFR or other evidence of chronic kidney damage, will continue to be exposed to additional risk factors for further progression of CKD, including hypertension, diabetes mellitus, recurrence of their underlying disease, nephrotoxic medications, and renal vascular disease. AKI is an important risk factor for short-term and long-term mortality, with a graded relationship between severity and mortality risk. Moreover, CKD with its subsequent hypertension and proteinuria independently increases the risk of adverse events, cardiovascular, and all-cause morbidity and mortality compounding the poor long-term outcomes among HCT patients. Continuous attention to even a modest decline in renal function is necessary in stem cell transplant survivors. To better delineate the incidence of CKD and risk factors for developing CKD following HCT, prospective studies are needed to better understand the epidemiology and pathophysiology of kidney dysfunction with the ultimate goal of targeted therapies that minimize the detrimental impact of this common complication. 

The number of HCTs performed continues to increase with improved success rates worldwide. An increase in CKD survivors who are at high risk for other chronic diseases is expected. The recognition of post-HCT CKD will continue to evolve and will need continued study. Physicians and public health officials will need to work in tandem to identify transplant patients at risk of developing CKD, thereby slowing the progression to ESRD and preventing associated chronic complications. Perhaps the creation of a national or regional registry may aid our understanding of acute and chronic kidney disease following HCT. 

## Conflict of interest 

Authors have no financial relationship to disclose. 

**Table 1.** Hematopoietic stem cell transplant procedures.

Type of cell transplanted	Blood source	Regimen	Is prophylaxis required for graft-versus-host?	Infectious prophylaxis
Autologous	Self or identical twin	Myeloablative	No	Antivirals for varicella zoster and herpes simplex virus. Fluconazole for fungi; trimethoprim-sulfamethoxazole for *Pneumocystis carinii* and toxoplasmosis
Allogeneic	HLA-matched related or unrelated donor, cord blood, HLA mismatched blood, haploidentical donor	Myeloablative or reduced intensity	Calcineurin inhibitors, methotrexate, mycophenolate mofetil, antithymocyte globulin, alemtuzumab, prednisone, sirolimus, cyclophosphamide	High-dose acyclovir, ganciclovir, or foscarnet for cytomegalovirus. Antivirals for varicella zoster and herpes simplex virus. Trimethoprim-sulfamethoxazole for Pneumocystis carinii and toxoplasmosis. Fluconazole, posaconazole, voriconazole, or micafungin for fungi.

**Table 2.** Etiologies and risk factors for acute kidney injury after hematopoietic stem cell transplant.

Etiology & risk factor	Potential mechanism	Comments
Acute GVHD	Acute kidney injury, intravascular volume depletion, cytokine release, inflammatory and immune-mediated injury	Both acute and chronic GVHD contribute to renal injury.
Post-transplant-related complications	Sepsis – systemic vasodilation and medication-related injury Sinusoidal obstruction syndrome – renal vasoconstriction	
Calcineurin inhibitors	Endothelial injury, renal vasoconstriction	Difficult to evaluate the role as it is given to many patients for prophylaxis against GVHD. Also interplay with drug synergism with antibiotics and antifungals.

GVHD = graft-versus-host disease.

**Table 3. Table3:** Etiologies and risk factors for chronic kidney injury after hematopoietic stem cell transplant.

Etiology & risk factor	Potential mechanism	Comments
GVHD	Acute kidney injury, intravascular volume depletion, cytokine release, inflammatory and immune-mediated injury	Both acute and chronic GVHD contribute to renal injury.
Thrombotic microangiopathy	Endothelial injury	
Glomerular disease Membranous nephropathy Minimal change disease FSGS IgA nephropathy	Endothelial injury, renal vasoconstriction	Membranous nephropathy most common. Heterogeneous studies regarding incidence. FSGS and IgA nephropathy mostly case reports.
Infection	Tubular injury and inflammation	Adenovirus associated with acute kidney injury.

GVHD = graft-versus-host disease; FSGS = focal segmental glomerulosclerosis; IgA = Immunoglobulin A.

**Table 4.** Evaluation and treatment of chronic kidney disease following hematopoietic cell transplantation.

Evaluation treatment	
Search for a specific diagnosis. Early referral to a nephrologist for disease comanagement should be considered. Disease severity should be assessed by a standardized definition of kidney function. Providers should attempt to estimate disease progression and potential complications. This includes an assessment of comorbidities. Specific evaluations to consider: – Glycemic control – Hypertensive management – Medication monitoring and toxicity assessments – Presence of proteinuria	Therapy should be based on specific diagnosis. – GVHD, TMA, CNI, infection, other causes Prevent and manage complications of decreased kidney function: – HTN – Anemia – Malnutrition – Medication dose adjustments – Detect drug interactions – Manage conditions that impair kidney function

GVHD = graft-versus-host disease; TMA = thrombotic microangiopathy; CNI = calcineurin inhibitor; HTN = hypertension.
